# Contrastive Speaker Representation Learning with Hard Negative Sampling for Speaker Recognition

**DOI:** 10.3390/s24196213

**Published:** 2024-09-25

**Authors:** Changhwan Go, Young Han Lee, Taewoo Kim, Nam In Park, Chanjun Chun

**Affiliations:** 1 Department of Computer Engineering, Chosun University, Gwangju 61452, Republic of Korea; chgo@chosun.ac.kr; 2 Intelligent Image Processing Research Center, Korea Electronics Technology Institute, Seongnam 13509, Republic of Korea; yhlee@keti.re.kr (Y.H.L.); kimtaewoo@keti.re.kr (T.K.); 3 Digital Analysis Division, National Forensic Service, Wonju 26460, Republic of Korea; naminpark@korea.kr

**Keywords:** speaker recognition, contrastive learning, hard negative sampling

## Abstract

Speaker recognition is a technology that identifies the speaker in an input utterance by extracting speaker-distinguishable features from the speech signal. Speaker recognition is used for system security and authentication; therefore, it is crucial to extract unique features of the speaker to achieve high recognition rates. Representative methods for extracting these features include a classification approach, or utilizing contrastive learning to learn the speaker relationship between representations and then using embeddings extracted from a specific layer of the model. This paper introduces a framework for developing robust speaker recognition models through contrastive learning. This approach aims to minimize the similarity to hard negative samples—those that are genuine negatives, but have extremely similar features to the positives, leading to potential mistaken. Specifically, our proposed method trains the model by estimating hard negative samples within a mini-batch during contrastive learning, and then utilizes a cross-attention mechanism to determine speaker agreement for pairs of utterances. To demonstrate the effectiveness of our proposed method, we compared the performance of a deep learning model trained with a conventional loss function utilized in speaker recognition with that of a deep learning model trained using our proposed method, as measured by the equal error rate (EER), an objective performance metric. Our results indicate that when trained with the voxceleb2 dataset, the proposed method achieved an EER of 0.98% on the voxceleb1-E dataset and 1.84% on the voxceleb1-H dataset.

## 1. Introduction

Speaker recognition refers to the technique of identifying a speaker from an input utterance. This is achieved by extracting features from the speech signal that are identifiable to the speaker, excluding linguistic information [1]. Various methods have been proposed for speaker recognition to extract features that can distinguish speakers; these features are called speaker representations. Speaker representation is a characteristic of how a speaker speaks, such as timbre, tone, and pronunciation, and can be utilized to perform speaker verification by calculating the similarity between speaker representations to determine whether the speaker is the same or not. In speech synthesis, speaker representation is utilized to synthesize the utterances of a speaker with linguistic information for a specific sentence [2]. Given that speaker recognition is employed for system security and authentication, it must extract a robust speaker representation to ensure a high recognition rate. Various machine learning and deep learning-based speaker recognition methods have been proposed for this purpose. Speech signals are high-dimensional vectors that encompass various factors beyond speaker features, including linguistic information. Approaches to speaker recognition research have been proposed to extract only a speaker’s unique features from speech signals and represent them in a low-dimensional vector. One of the machine learning-based speaker recognition approaches uses a Gaussian mixture model (GMM) to identify the speaker from the speech signal based on the probability density distribution, in which the speech features modeled by the Gaussian distribution are called GMM supervectors [3]. Although the GMM supervector contributes to extracting speaker features, it is limited because a high-dimensional Gaussian distribution must be represented to model the speech signal. Therefore, dimensionality reduction methods, such as principal component analysis (PCA) and linear discriminant analysis (LDA), have been utilized to project speech signal into low-dimensional vector, and the extracted feature was called i-vector [4]. Although the i-vector can extract detailed features of speech signals, it still contains many factors beyond the speaker’s features.

To overcome these limitations, approaches utilizing deep learning models assume that the embedding extracted from the layer of the model that identify speakers have robust features that allow speaker-to-speaker discrimination; the embedding extracted through this process was called d-vector [5]. The d-vector was the first deep learning-based speaker representation that achieved high performance by inputting frame-level features extracted from speech signals into deep neural networks such as convolutional neural networks (CNNs) and recurrent neural networks (RNNs) [6]. Furthermore, to extract statistical features from speech signal, a time-delay neural network (TDNN) [7], a model designed based on a 1-D convolutional layer, and statistical pooling were used to extract fixed-length embedding using the mean and variance of frame-level feature, called the x-vector [8]. After training a deep learning model as a speaker identification model, such as the d-vector, the x-vector utilizes embedding extracted from specific layer. Recently, a pooling method based on the attention mechanism has been proposed to emphasize a speaker’s unique features for the frame and time domains [9], and studies have been proposed to enhance the robustness of speaker feature extraction by advancing model architectures [10]. As a representation example, DF-ResNet was proposed by extending the number of layers in the ResNet architecture to create a deeper ResNet [11]. Additionally, various methods, such as the progressive channel fusion (PCF), which extracts features by splitting them into branches and then combining them, have been proposed [12].

Because speaker recognition determines speaker matching based on the similarity between extracted speaker representations, methods have been proposed using contrastive learning to distinguish the boundaries between different classes using the Euclidean distance or cosine similarity between representations [13,14]. When data in the same class are called positive, and data in a different class are called negative, contrastive learning maximizes the similarity between positives and minimizes the similarity between negatives. For representative contrastive learning methods, there is contrastive loss, which performs learning on either positive or negative relationships, and triplet loss which performs contrastive learning considering both positive and negative relationships [15]. One speaker recognition method that utilizes such contrastive learning is supervised contrastive learning, which learns the speaker’s relationship between representations. One of the most widely utilized supervised contrastive learning methods in recent years is information noise contrastive estimation (InfoNCE), which shows high performance by considering one-to-many relationships instead of only one-to-one or one-to-two relationships, such as contrastive loss and triplet loss [16]. InfoNCE is a loss function that performs contrastive learning through cross-entropy loss by inputting *N* positive pairs with different classes into the model and estimating the similarity of *N* × *N* using the inner product between the extracted representations. A representative study utilizing InfoNCE is contrastive language-image pretraining (CLIP), which utilizes a large dataset of image–text pairs to learn the relationship between images and text [17]. To overcome the limitation that conventional image classification methods may be limited in generalization, because they optimize only within a limited set of categories, CLIP performs zero-shot classification, which classifies the category to which the input data belongs without the restriction of labels and demonstrates its effectiveness in supervised contrastive learning by achieving high performance, including achieving state-of-the-art performance in various classification tasks [18]. Another method for utilizing contrastive learning is self-supervised contrastive learning, which uses data without labeling to extract features [19,20,21]. Self-supervised contrastive learning can extract speaker features from speech signals by using only data without labeling, such as slicing a clip of the waveform domain to perform learning between segments or performing reconstruction learning to recover the masked parts after masking the extracted frame-level features. However, self-supervised contrastive learning methods tend to perform worse than supervised learning-based speaker recognition methods, because their features limit them [22]. In this way, contrastive learning can be applied to perform mapping between different modalities such as text, images, and audio. It is also an efficient training method that can be utilized in various studies, including image searches, natural language processing, recommendation systems, speech recognition, and medical image analyses, by training the similarities between data to generate effective representations and applications.

The most important issue in contrastive learning is to consider a hard negative sample, which comprises data that are negative, but have extremely similar features to the positive and are easily mistaken for positive. Because hard negative samples can be a factor in reducing recognition rates, various countermeasures have been proposed to minimize the similarity between hard negative pairs, and these countermeasures are called hard negative sampling. One of the representative hard negative sampling methods in speaker recognition is the generalized end-to-end loss (GE2E) [23], which estimates the hardest negative speaker among the registered speakers for an input utterance and then minimizes the similarity with the utterance of the hard negative speaker. By focusing on hard negative samples, GE2E has achieved high performance in speaker recognition between hard negative pairs; however, to estimate the hard negative speaker for an input utterance, a representation of all registered speakers’ utterances must be extracted, which requires using a pretrained speaker recognition model.

The most widely utilized hard negative sampling strategy to overcome this limitation involves estimating a hard negative sample by measuring the similarity between representations in mini-batches during contrastive learning [24]. A representative method that utilizes this is bootstrapping language-image pretraining (BLIP) [25], which performs hard negative sampling on image–text pairs. BLIP has demonstrated the effectiveness of hard negative sampling by achieving higher performance than CLIP and a large scale image and noisy-text embedding (ALIGN) [26], which perform contrastive learning on image–text pairs without hard negative sampling.

This paper proposes a framework for training a robust speaker recognition model through contrastive learning with hard negative sampling. Specifically, the proposed framework estimates hard negative samples in a mini-batch by comparing the similarities between speaker representations extracted from deep learning models during contrastive learning. It then performs binary classification to determine whether the relationship between speaker representations is positive or hard negative, using a cross-attention mechanism [27]. To demonstrate the performance of the proposed method, we compared it with the conventional method using three models: ResNet34 [28], extended time-delay neural network (E-TDNN) [29], and emphasized channel attention, propagation, and aggregation in TDNN (ECAPA-TDNN) [30]. We evaluated the performance using the objective metric of equal error rate (EER) in terms of speaker verification on the voxceleb1-E and voxceleb1-H dataset [31]. The remainder of this paper is structured as follows: Section 2 discusses related work, Section 3 presents the proposed method, Section 4 describes the experiments and results, and Section 5 concludes with final remarks.

## 2. Related Works

**Figure 1:**
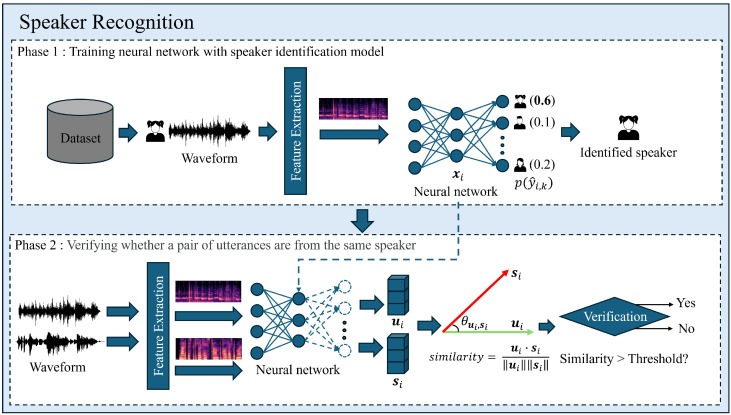
Overview of a representative deep learning-based speaker recognition framework. Phase 1 illustrates the process of training the neural network into a model that identifies speakers, and Phase 2 illustrates the process of utilizing the neural network trained in Phase 1 to determine whether a pair of utterances are from the same speaker or not. $x_{i}$ represents the embedding vector for input data, and $p({\hat{y}}_{i,k})$ denotes the probability that the input data belongs to the *k*-th class. $u_{i}$ and $s_{i}$ represent the speaker representation extracted from a pair of utterances.

Figure 1 illustrates an overview of representative deep learning-based speaker recognition frameworks. In Phase 1 illustrates the process of training a neural network that identifies speakers using a large number of datasets, like the voxceleb2 dataset [32], which consisted of 5994 speakers. In this process, using features extracted from the input utterance as input, the neural network is trained on which of the registered speakers the input utterance is most resemble. While cross-entropy loss can be utilized as a loss function for learning a speaker identification model in this manner, Deng et al. proposed additive angular margin softmax (AAMSoftmax), a loss function that imposes a margin-like penalty between classes, to overcome the limitation that cross-entropy loss learns to optimize the data to the center of the class, but does not explicitly optimize the representation between classes [33]. AAMSoftmax has been shown to improve recognition rates in various recognition tasks significantly, and is particularly robust when utilizing deep neural networks, such as ResNet [34,35]. The equations for the cross-entropy loss and AAMSoftmax are as follows:

(1) $$L_{\mathrm{CE}}=-\sum_{i=1}^{N}\sum_{k=1}^{K}y_{i,k}\log\left(p\left({\hat{y}}_{i,k}\right)\right)$$

(2) $$\langle w_{i},x_{j}\rangle =\frac{w_{i}\cdot x_{j}}{∥w_{i}∥∥x_{j}∥}$$

(3) $$L_{\mathrm{AAM}}=-\sum_{i=1}^{N}\log\frac{e^{s(\langle w_{y_{i}},x_{i}\rangle +m)}}{e^{s(\langle w_{y_{i}},x_{i}\rangle +m)}+\sum_{k=1,k\neq y_{i}}^{K}e^{s(\langle w_{k},x_{i}\rangle )}}$$

Equation (1) shows the formula for the cross-entropy loss. *N* is the number of mini-batches, and $y_{i,k}$ is the ground truth in a one-hot label. *K* represents the number of classes, $p({\hat{y}}_{i,k})$ is the probability that the input data belong to the *k*-th class. The cross-entropy loss aims to maximize the probability value of the ground truth for the data while minimizing the probability value for the other classes. However, AAMSoftmax calculates the angle between the class-wise weight and $x_{i}$, the embedding of the input data extracted from the model, as shown in Equation (2), and as shown in Equation (3), optimizes to maximum the cosine similarity between the ground truth weight $w_{y_{i}}$ and $x_{i}$. *m* is the angular margin, and *s* is the scale value. AAMSoftmax aims to create explicit boundaries between classes by providing a margin. The embeddings extracted from a specific layer of a neural network trained to identify a large number of speakers using the above loss functions have robust speaker-distinguishable features and are used as speaker representations.

Because speaker verification relies on measuring the similarity between speaker representations, another approach to training a speaker recognition model can utilize contrastive learning. Contrastive learning maximizes the similarity between the same targets in an embedding space while minimizing the similarity between different targets. Speaker recognition can utilize contrastive learning to maximize the similarity between positive speaker representations and minimize the similarity between negative speaker representations; representative contrastive learning methods include contrast loss, which focuses on either positive or negative relationships between a pair of data, and triplet loss, which considers both positive and negative cases simultaneously. Among these, one of the popular methods is a InfoNCE, which trains considers all relationships between all the data in mini-batch size. InfoNCE is a contrastive learning method that utilizes cross-entropy loss to train relationships between *N* pairs with different classes. When denoting each extracted representation in the model as $u_{i}$ and $s_{i}$, InfoNCE performs contrastive learning by considering two-aspects relationships between $u_{i}$ and $s_{i}$, and between $s_{i}$ and $u_{i}$. For representative examples of utilizing InfoNCE, there are contrastive visual representation learning from text (ConVIRT) [36] and CLIP, which learns the relationship between image and text, and between text and image through InfoNCE for learning about multi-modality. The equations for InfoNCE that consider both aspects of the representation can be expressed as follows:

(4) $$\begin{array}{c} L_{\mathrm{InfoNCE}}=-\sum_{i=1}^{N}y_{i}\left(\log\frac{e^{\left(\langle u_{i},s_{i}\rangle /\tau \right)}}{\sum_{j=1}^{N}e^{\left(\langle u_{i},s_{j}\rangle /\tau \right)}}+\log\frac{e^{\left(\langle s_{i},u_{i}\rangle /\tau \right)}}{\sum_{j=1}^{N}e^{\left(\langle s_{i},u_{j}\rangle /\tau \right)}}\right) \end{array}$$

Equation (4) shows the formula for InfoNCE. τ is a learnable temperature parameter that scales the two inner values. InfoNCE has recently been utilized in many contrastive learning studies because of its advantage in considering one-to-many relationships rather than only one-to-one or one-to-two relationships, such as conventional contrastive loss and triplet loss [37,38]. InfoNCE has also recently been applied as a hard negative sampling strategy, because it can estimate hard negative samples within mini-batches based on the estimated similarity [39].

After training a neural network to extract speaker representations with the above approaches, in Phase 2, it performs speaker verification by extracting speaker representations from the input utterances using the trained neural network. For a pair of utterances, the representations, extracted as $u_{i}$ and $s_{i}$, respectively, speaker verification computes a cosine similarity from the extracted representations, based on a threshold: if it is higher, the two utterances are from the same speaker, and if it is lower, the utterances are from different speakers.

In this paper, cross-entropy, AAMSoftmax, and InfoNCE were used as baselines to demonstrate the performance of the proposed method. For AAMSoftmax, we set the margin to 0.2 and the scale to 30.

## 3. Contrastive Speaker Representation Learning Framework

**Figure 2:**
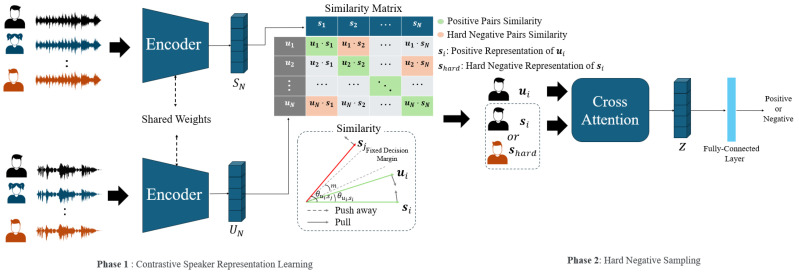
Overview of the proposed speaker representation learning framework. The input to the encoder is positive pairs consisting of different speakers, and each encoder has the same architecture and shares the same weight. *N* denotes the size of mini-batches, $U_{N}$ and $S_{N}$ denote the *N* speaker representations extracted by each encoder, and *m* denotes the margin value. Phase 1 illustrates the process of contrastive learning for training speaker relationship between *N* positive pairs of utterances from the same speaker and $N^{2}-N$ negative pairs of utterances from different speakers. Phase 2 illustrates the process of performing hard negative sampling using a cross-attention mechanism based on a similarity matrix. $s_{hard}$ denotes the hard negative sample for $u_{i}$ estimated through Phase 1, and Z denotes the result of the cross-attention mechanism.

In this paper, we propose a framework to train the speaker relationship between representations through contrastive learning with hard negative sampling. Figure 2 illustrates an overview of the proposed framework. *N* denotes the size of the mini-batch. The input to speaker encoder is *N* positive pairs consisting of different speakers, and each encoder outputs speaker representations $U_{N}$ and $S_{N}$. Each encoder had the same architecture, and shared the weight. The similarity matrix computes all representations of N×N similarities between $u_{i}$ and $s_{i}$. We aimed to develop a robust speaker recognition model using hard negative sampling based on the estimated similarities. The proposed framework comprises contrastive speaker representation learning and hard negative sampling.

### 3.1. Contrastive Learning with Hard Negative Sampling

Phase 1 illustrates the process of contrastive speaker representation learning. In this paper, the similarity was calculated using the cosine similarity between two representations extracted when learning the speaker relationship between representations, and contrastive learning was performed to minimize the similarity between negative pairs by assigning a margin. This process is called additive angular margin information noise contrastive estimation (AAM-InfoNCE), and the equations for AAM-InfoNCE can be expressed as follows:

(5) $$\begin{array}{cc} \; & L_{\mathrm{AAM-InfoNCE}}=\\ \; & -\sum_{i=1}^{N}y_{i}(\log\frac{e^{s(\left(\langle u_{i},s_{i}\rangle +m\right)/\tau )}}{e^{s(\left(\langle u_{i},s_{i}\rangle +m\right)/\tau )}+\sum_{j=1,j\neq i}^{N}e^{s(\langle u_{i},s_{j}\rangle /\tau )}}\\ \; & \;\;\;+\log\frac{e^{s(\left(\langle s_{i},u_{i}\rangle +m\right)/\tau )}}{e^{s(\left(\langle s_{i},u_{i}\rangle +m\right)/\tau )}+\sum_{j=1,j\neq i}^{N}e^{s(\langle s_{i},u_{j}\rangle /\tau )}}) \end{array}$$

Equation (5) is the overall formula of AAM-InfoNCE, which aims to learn to minimize the angle between $u_{i}$ and $s_{i}$, a positive pair, and maximize between $u_{i}$ and $s_{j}$, a negative pair, by offering a margin to the angle between $u_{i}$ and $s_{j}$ to form a boundary, as shown in Figure 2. In this paper, we perform contrastive learning considering the two aspects of $u_{i}$ and $s_{i}$ and $s_{i}$ and $u_{i}$, as shown in Equation (4), and set the margin value to 0.2 and the scale value to 30.

Phase 2 involves calculating the relationship between the query and key with different values as attention weights between 0 and 1 using softmax, and then performing hard negative sampling using a cross-attention mechanism that conveys the relationship between the query and key to the value through the inner product between the value and attention weight. The motivation for this approach is to align the image and text representations before fusing (ALBEF) [40], which trains the relationship between the image–text through contrastive learning, hard negative sampling, and masked language modeling to predict the masked text token. ALBEF performs hard negative sampling by calculating the attention weight between representations extracted from hard negative image–text pairs through a cross-attention mechanism and performing a binary classification of whether the image–text pair is positive or hard negative. This method is utilized in various fields, such as dataset filtering and retrieval, and it stands out because it can perform effective contrastive learning by focusing on the relationship between hard negative pairs [25,39]. In this paper, in the hard negative sampling process, inputs positive pairs or $u_{i}$ and $s_{\mathrm{hard}}$ that hard negative sample of $u_{i}$ or $s_{i}$ and $u_{\mathrm{hard}}$ that a hard negative sample of $s_{i}$ as query, key, and value, and Z, extracted through the cross-attention mechanism, is inputted into the fully connected layer to perform binary classification of the speaker agreement of the relationship between the pair of representations.

### 3.2. Speaker Encoder

**Figure 3:**
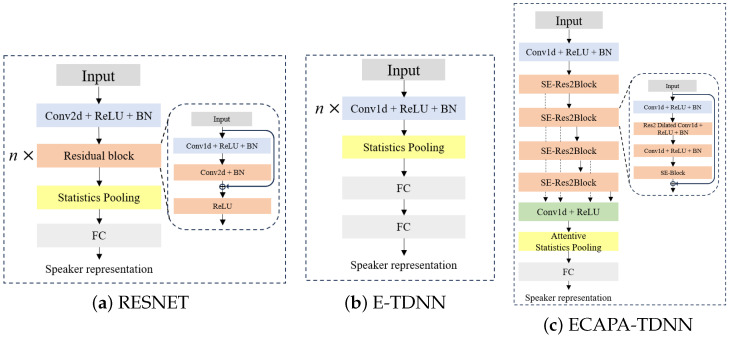
Architecture of the speaker encoders. *n* denotes the number of layers. (**a**) is the architecture of ResNet, which is widely utilized in speaker recognition and replaces the conventional average pooling layer with a statistics pooling layer. (**b**) is the architecture of E-TDNN, which extends the number of filters in the convolutional layer in the TDNN consisting of three components: 1-D convolutional layer, statistics pooling layer, and fully connected layer. (**c**) is the architecture of ECAPA-TDNN, which enhances the TDNN architecture using a squeeze-and-excitation block, Res2Net modules, and an attention mechanism.

Figure 3 shows the architecture of the speaker encoder utilized in this paper, which utilizes three model architectures widely utilized in speaker recognition: ResNet, E-TDNN, and ECAPA-TDNN.

Figure 3a shows the ResNet architecture. ResNet’s architecture, which is widely utilized in speaker recognition, is an r-vector structure that replaces the conventional average pooling layer with a statistical pooling layer [41]. The r-vector outputs a fixed-length speaker representation through statistical pooling, utilizing frame-by-frame averages and variances for channel and frequency. This paper used the ResNet34 architecture to extract 256-dimensional speaker representations.

Figure 3b illustrates the architecture of the E-TDNN, which expands the number of filters in the convolutional layers within the TDNN framework. This enhancement facilitates extracting features from time-series data via three key components: a 1-D convolutional layer, statistics pooling, and a fully connected layer. In addition, the E-TDNN extracts dilated features from time series data using 1-D convolutional layers with various kernel sizes; the E-TDNN utilized in this paper consisted of four 1-D convolutional layers with 1500 filters, and the size of each kernel was configured as {5, 5, 3, 3}. It includes a statistical pooling layer and two fully connected layers designed to extract a 192-dimensional representation of the speaker.

Figure 3c shows the architecture of the ECAPA-TDNN, which consists of a squeeze-and-excitation block [42], which compresses data to retain temporal information and emphasize the channel, and a squeeze-and-excitation Res2Block (SE-Res2Block), which utilizes Res2Net modules [43] to divide each channel into several branch channels and extract features for each branch channel through group convolution. ECAPA-TDNN also utilizes attentive statistics pooling, which performs statistical pooling after computing the weighted mean and weighted standard deviation of the features on a frame-by-frame basis through a channel-dependent self-attention mechanism [44] of the extracted features. The channel size of the ECAPA-TDNN used in this paper was 1024, and a 192-dimensional speaker representation was extracted.

As inputs for all speaker encoders, we used 80-dimensional mel spectrograms extracted through a short-time Fourier transform (STFT) utilizing a mel-filter bank. We used Adam [45] as the optimization function for learning, and set the learning rate to 0.001. We decreased the learning rate by 0.97 times for every 1 epoch, and performed 300 epochs in total. For the size of the mini-batch, we set 256, and for data augmentation, we used the MUSAN dataset [46], which consists of noises such as babble, speech, and music, and utilized SpecAugment, which performs masking with a size of 0–10 in the time domain and 0–8 in the frequency domain [47]. We also set the number of heads of the cross-attention mechanism to eight when performing hard negative sampling and set the dimension of the fully connected layer to 2 for binary classification.

## 4. Performance Evaluation

In this paper, we trained the models using cross-entropy loss, AAMSoftmax, InfoNCE, and the proposed framework to compare their performance in terms of speaker verification for three models: ResNet34, E-TDNN, and ECAPA-TDNN. For InfoNCE and AAM-InfoNCE, we compared their performance with and without hard negative sampling. We used the voxceleb2 dataset for training, a large dataset consisting of 1,092,009 utterances from 5994 speakers. During training, we used a 2 s cutoff for utterances, slicing for shorter utterances, and zero-padding for longer utterances. The sampling rate was 16 kHz, and the window size and hop length were set to 512 and 256, respectively. As evaluation datasets, we used the voxceleb1-E dataset, which consists of 581,480 pairs of utterances for speaker verification by randomly extracting utterances from the voxceleb1 dataset and the voxceleb1-H dataset, which consists of 552,536 hard negative pairs of the same nationality and gender [48].

As an evaluation metric, we used the EER, which represents the point at which the values of the false acceptance rate (FAR) and false rejection rate (FRR) become equal. FAR is the rate at which a negative is mistaken for a positive, while FRR is the rate at which a positive is mistaken for a negative. The FAR and FRR can be expressed as follows:

(6) $$\mathrm{FAR}=\frac{\mathrm{FP}}{\mathrm{FP}+\mathrm{TN}}$$

(7) $$\mathrm{FRR}=\frac{\mathrm{FN}}{\mathrm{FN}+\mathrm{TP}}$$

Equations (6) and (7) represent the formulas for calculating FAR and FRR. When calculating the EER, the ROC curve is utilized to calculate the FAR and FRR at various thresholds ranging from 0 to 1. The EER is the value at the point where the two curves intersect [49]. To improve EER performance, adaptive score normalization (AS-Norm) can be utilized, which improves recognition rates by consistently normalizing the score distributions between the enrollment and test utterances. In this paper, we perform AS-Norm by extracting speaker-wise representations, which are the averages of the speaker representations extracted from all utterances of each speaker in the training dataset. During evaluation, for the cosine similarity of speaker representations between the enrollment and test utterances in voxceleb1-E, score normalization is performed using the mean and variance of the top-400 cosine similarities between the speaker representation of the enrollment utterances and the speaker-wise representations [50].

Table 1 lists the performances of each loss function in ResNet34. When trained with cross-entropy loss, voxceleb1-E shows an EER of 1.54%, and voxceleb1-H shows an EER of 2.82%, respectively. When trained with InfoNCE, voxceleb1-E showed an EER of 1.37%, and voxceleb1-H showed an EER of 2.64%, indicating that InfoNCE outperformed cross-entropy loss trained with classification methods by 0.17% and 0.18%, respectively. We also confirmed that learning with InfoNCE by performing hard negative sampling resulted in a small performance improvement of 0.01% for voxceleb1-E, but a 0.29% improvement for voxceleb1-H. Furthermore, training with AAMSoftmax showed EER of 1.29% and 2.25% on each dataset, whereas training with AAM-InfoNCE showed a 0.05% performance improvement on voxceleb1-E but a 0.1% performance drop on voxceleb1-H over AAMSoftmax. However, by training with AAM-InfoNCE, which implements hard negative sampling, we observed a 0.12% enhancement in performance on voxceleb1-H compared to ResNet34 trained with AAMSoftmax. This resulted in an EER of 1.19% on voxceleb1-E and 2.13% on voxceleb1-H for each dataset.

Table 2 lists the performance of each loss function for E-TDNNs. When training with cross-entropy loss, voxceleb1-E showed an EER of 1.82% and voxceleb1-H showed an EER of 2.99%, whereas when training with InfoNCE, voxceleb1-E showed an EER of 1.71% and voxceleb1-H showed an EER of 2.89%, which is a performance improvement of 0.11% and 0.1%, respectively. For InfoNCE with hard negative sampling, we confirmed a performance improvement of 0.15% for voxceleb1-E and 0.34% for voxceleb1-H, with EER of 1.67% for voxceleb1-E and 2.65% for voxceleb1-H. When trained with AAMSoftmax, we confirmed EER of 1.44% and 2.39% on each dataset, and when trained with AAM-InfoNCE, we confirmed a performance drop of 0.11% on voxceleb1-E and a 0.03% improvement on voxceleb1-H compared to AAMSoftmax. However, when trained with AAM-InfoNCE with hard negative sampling, we confirmed a similar performance on voxceleb1-H compared with AAMSoftmax.

Table 3 lists the performance of each loss function for ECAPA-TDNN. When training with cross-entropy loss, voxceleb1-E showed an EER of 1.49%, and voxceleb1-H showed an EER of 2.51%, while when training with InfoNCE, voxceleb1-E showed an EER of 1.37% and voxceleb1-H showed an EER of 2.34%, which were 0.12% and 0.17% better than cross-entropy loss, respectively. For InfoNCE with hard negative sampling, we confirmed a performance improvement of 0.29% for voxceleb1-E and 0.42% for voxceleb1-H, with EER of 1.20% for voxceleb1-E and 2.09% for voxceleb1-H. When trained with AAMSoftmax, the EERs were 1.10% and 2.11% for each dataset, respectively. When trained with AAM-InfoNCE, the performance improvement over AAMSoftmax was 0.09% for voxceleb1-E and 0.18% for voxceleb1-H. In addition, when trained with AAM-InfoNCE performing hard negative sampling, the best performance was achieved on voxceleb1-E and voxceleb1-H with EERs of 0.98% and 1.84%, respectively, among the three models. The performances of the three models for each loss function demonstrate that the proposed method with hard negative sampling is more robust than the conventional training method for hard negative pairs.

As an additional experiment, we visualized the embedding space of the ECAPA-TDNN using t-distributed stochastic neighbor embedding (t-SNE) [51], where higher similarity corresponds to closer distances between representations, and lower similarity corresponds to farther distances. We further compared and analyzed the performance of AAM-InfoNCE depending on size of the margins and mini-batch sizes.

**Figure 4:**
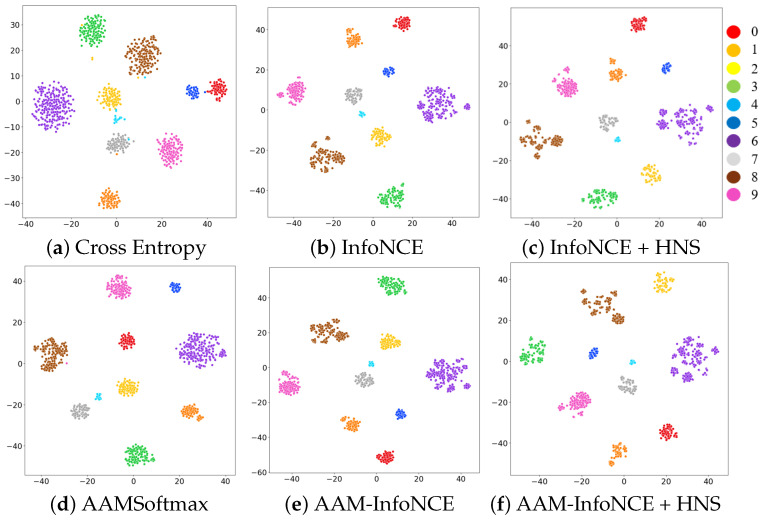
t-SNE analysis of the speaker representations of 10 unseen speakers extracted through ECAPA-TDNN. We visualized the embedding space of ECAPA-TDNNs trained with cross-entropy, InfoNCE, AAMSoftmax, and AAM-InfoNCE, respectively. The t-SNE visualizes the embedding space in a way that embeddings with higher similarity are closer, while those with lower similarity are farther. Each color represents the identity of each speaker’s utterance.

Figure 4 illustrates the t-SNE of the extracted representation after training the ECAPA-TDNN with each loss function for 10 unseen speakers, where each color represents the identity of each speaker’s utterance. When we visually analyzed the results regarding the embedding space, we confirmed that Figure 4a was the least compact among the loss functions, but the compactness of the remaining loss functions was similar. Therefore, for further analysis through objective metrics, we utilized homogeneity and completeness scores, commonly utilized metrics in clustering [52].

Table 4 shows the homogeneity and completeness scores for the t-SNE results of 10 unseen speakers. To perform both evaluation metrics, we assume that the results of t-SNE are the results of k-means clustering [53] with k = 10, and perform a performance evaluation on whether the labels of the predicted results using the k-means algorithm and the labels of the actual representation are consistent. The labels for the unseen speaker were assigned labels from 0 to 9 for each color, as shown in Figure 4, and the equations for homogeneity and completeness are as follows:

(8) $$\mathrm{homogeneity}=1-\frac{H(C|K)}{H(C)}$$

(9) $$\mathrm{completeness}=1-\frac{H(K|C)}{H(K)}$$

Equations (8) and (9) present the formulae for calculating the homogeneity and completeness scores. H(C|K) denotes the conditional entropy of the ground-truth label for the predicted label of a given k-means cluster, and H(C) denotes the entropy of the ground-truth label. H(K|C) is the conditional entropy of the predicted label for the true label and H(K) denotes the entropy of the predicted label. Analyzing the results in Table 4, we confirmed that the ECAPA-TDNN trained by AAM-InfoNCE with hard negative sampling performed the best, with a homogeneity score of 0.98 and a completeness score of 0.937.

Table 5 presents a comparative analysis of the performance of AAM-InfoNCE for three different margin values (0.1, 0.2, and 0.3) to analyze the impact of the margin size on performance. These experiments are conducted to determine whether the margin value significantly affects performance and to find the optimal value. The value of the scale was fixed at 30 when measuring the performance, and training was performed using the same training parameters. In conclusion, on the voxceleb1-H dataset, AAM-InfoNCE performs more robustly against hard negative pairs when performing hard negative sampling, regardless of the value of the margin. when the margin is 0.1 we confirmed with an EER of 1.07% for voxceleb1-E and 1.91% for voxceleb1-H, When the margin was 0.2, we confirmed the best with an EER of 0.98% on voxceleb1-E, and when the margin was 0.3, the best with an EER of 1.78% on voxceleb1-H. As a result, the performance at a margin value of 0.2 was 0.14 EER better compared to a margin value of 0.1, demonstrating that the proper setting of the margin value has a impact on the results.

The mini-batch size can affect performance because the proposed method considers more negative samples as the mini-batch size increases [54]. In addition, because the hard negative sampling process assumes that the most similar sample among the negative samples in the mini-batch is the hard negative sample, the larger the mini-batch size, the more likely it is to extract a hard negative sample. Therefore, the mini-batch size significantly affects the performance of the proposed method. The GPU used in this paper is NVIDIA A5000, and Table 6 shows the results from 32 to 256, the maximum batch size that can be allocated to it. The smallest batch size, 32, showed the lowest performance, with performances of 1.21% for voxceleb1-E and 2.19% for voxceleb1-H. However, the performance tended to improve as the mini-batch size increased, with the largest batch size of 256 performing the best with performances of 0.98% for voxceleb1-E and 1.84% for voxceleb1-H.

In our experiments, we confirmed that the proposed method outperforms most conventional approaches, particularly on the voxceleb1-H dataset, which includes pairs of hard negative utterances. We attribute this performance improvement to our contrastive learning method, which not only considers a large number of negative samples, but also identifies the hardest negative samples and reduces their similarity through hard negative sampling, contributing significantly to the overall results. However, the method has a limitation: the margin and value must be set carefully, and since performance can fluctuate based on the mini-batch size, larger mini-batches may require substantial resources.

## 5. Conclusions and Future Work

Our study introduced a framework for developing robust speaker recognition models using contrastive learning with hard negative sampling. To demonstrate the performance of our proposed method, we compared the performance of speaker encoders trained with conventional loss functions and trained with the proposed framework. As a result, we confirmed that our proposed method had superior performance on the voxceleb1-E and voxceleb1-H datasets than the conventional methods. We also confirmed that the proposed method performed more robustly when trained with a larger mini-batch size; the more negative samples could be considered. Because conventional speaker recognition methods have the limitation of focusing on timbre information while ignoring alignment, we would like to develop a method that can consider alignment in the future.

## Figures and Tables

**Table 1:** Performance comparison of ResNet34 for each training method on EER. HNS means hard negative sampling, and analyzes performance on whether it is performed.

Model	Method	HNS (o/x)	EER (%)	
			Voxceleb1-E	Voxceleb1-H
ResNet34	Cross Entropy	x	1.54	2.82
	InfoNCE	x	1.37	2.64
		o	1.36	2.35
	AAMSoftmax	x	1.29	2.25
	AAM-InfoNCE (Proposed)	x	1.24	2.35
		o	1.19	2.13

**Table 2:** Performance comparison of E-TDNN with each training method on EER.

Model	Method	HNS (o/x)	EER (%)	
			Voxceleb1-E	Voxceleb1-H
E-TDNN	Cross Entropy	x	1.82	2.99
	InfoNCE	x	1.71	2.89
		o	1.67	2.65
	AAMSoftmax	x	1.44	2.39
	AAM-InfoNCE (Proposed)	x	1.55	2.42
		o	1.52	2.38

**Table 3:** Performance comparison of ECAPA-TDNN with each training method on EER.

Model	Method	HNS (o/x)	EER(%)	
			Voxceleb1-E	Voxceleb1-H
ECAPA-TDNN	Cross Entropy	x	1.49	2.51
	InfoNCE	x	1.37	2.34
		o	1.20	2.09
	AAMSoftmax	x	1.10	2.11
	AAM-InfoNCE (Proposed)	x	1.01	1.93
		o	0.98	1.84

**Table 4:** Homogeneity and completeness score comparative analysis of ECAPA-TDNN on t-SNE.

Method	HNS (o/x)	Homogeneity	Completeness
Cross Entropy	x	0.939	0.890
InfoNCE	x	0.957	0.907
	o	0.966	0.935
AAMSoftmax	x	0.968	0.920
AAM-InfoNCE (Proposed)	x	0.972	0.926
	o	0.980	0.937

**Table 5:** Performance analysis of ECAPA-TDNN trained with AAM-InfoNCE depending on the size of the margin on EER.

Size of Margin	HNS (o/x)	EER (%)	
		Voxceleb1-E	Voxceleb1-H
0.1	x	1.12	1.99
	o	1.07	1.91
0.2	x	1.01	1.93
	o	0.98	1.84
0.3	x	1.05	1.97
	o	1.02	1.78

**Table 6:** Performance analysis of ECAPA-TDNN trained with AAM-InfoNCE performing hard negative sampling depending on the mini-batch size on EER.

Batch Size	EER (%)	
	Voxceleb1-E	Voxceleb1-H
32	1.21	2.19
64	1.16	2.21
128	1.07	1.99
256	0.98	1.84

## Data Availability

Data are contained within the article.
